# Uptake of and intention to use oral pre-exposure prophylaxis for HIV among pregnant and post-natal women in Eswatini: a cross-sectional survey

**DOI:** 10.3389/frph.2023.1253384

**Published:** 2023-10-27

**Authors:** Philisiwe Ntombenhle Khumalo, Siphiwesihle Sibonisiwe Mkhonta, Kikanda Kindandi, Sindy Matse, Phinda Brian Dlamini, Vincent Tukei, Rhoderick Machekano, Godfrey Woelk

**Affiliations:** ^1^ Strategic Information and Evaluation/Clinical Services Delivery Department, Elizabeth Glaser Pediatric AIDS Foundation, Mbabane, Eswatini; ^2^ Eswatini National AIDS Program, Ministry of Health, Mbabane, Eswatini; ^3^ Research, Elizabeth Glaser Pediatric AIDS Foundation, Washington, DC, United States

**Keywords:** pregnant and post-natal women, HIV pre-exposure prophylaxis, PrEP, uptake, intention, Eswatini

## Abstract

**Introduction:**

In Eswatini, HIV incidence among women of childbearing age is 1.45%. Eswatini introduced oral pre-exposure prophylaxis (PrEP) for HIV prevention in 2016 and requires that all HIV-negative pregnant and post-natal women (PPW) visiting health care facilities be offered PrEP.

**Methods:**

Between September-November 2021, we conducted a survey among HIV-negative PPW from 16 purposively selected healthcare facilities in the Hhohho and Shiselweni regions in Eswatini. We interviewed consenting HIV-negative PPW using a structured questionnaire to collect data on PrEP knowledge, attitudes, intentions, and practices, as well as information on partner HIV status and stigma. Multivariate logistic regression was used to determine predictors of PrEP use and intention, adjusted for significant covariates.

**Results:**

Of 1,484 PPW women approached, 1,149 consented and were interviewed, of whom 704 (61.3%) were post-partum and 445 (38.7%) pregnant. The median age was 25 years [Interquartile Range (IQR) = 21–30 years], with 533 (46.4%) 18–24 years old. Among the 1,149 women, 930 (80.7%) had ever heard about PrEP; 635 (55.3%) had knowledge about PrEP; 183 (15.9%) were currently using PrEP; and 285 (24.8%) had ever used PrEP. Increased odds of PrEP use were associated having HIV-positive male partner (aOR:7.76, 95%CI 3.53- 17.04); positive attitudes to PrEP (aOR:1.56, 95%CI: 1.02–2.40); and high self-efficacy (aOR:1.49, 95%CI:1.13–1.98). Among 864 women who never used PrEP, 569 (65.3%) intended to use PrEP in the future. Odds of intention to use PrEP were higher among women with low levels of education (aOR:2.23, 95% CI: 1.32–3.77); who ever heard about PrEP (aOR:1.69, 95%CI: 1.12–2.56); and had high self-efficacy (aOR:1.57, 95%CI: 1.31–1.87). Regarding stigma, among all women, 759 (66%) either agreed or strongly agreed that people would think they have HIV if they were to use PrEP; 658 (57.3%) reported they would be labelled as having multiple sex partners; 468 (40.7%) reported that their partner would think they are having risky sex with other people. Of 102 women who had discontinued PrEP, a majority stopped due to side effects 32 (35.2%).

**Conclusion:**

Only about 50% of women had knowledge of PrEP, and PrEP uptake among PPW was low, though intention to use appeared high. More efforts to reduce stigma and promote PrEP use, including adequate information on side effects, are needed.

## Introduction

In 2016, the World Health Organization (WHO) strongly recommended offering oral pre-exposure prophylaxis (PrEP) containing tenofovir disoproxil fumarate (TDF) and emtricitabine (FTC), as an additional prevention choice for people at substantial risk of HIV infection as part of combination HIV prevention (1). Oral PrEP reduces the risk of HIV acquisition if used correctly as part of a combination prevention strategy. Several controlled trials have provided rigorous evidence that oral daily PrEP is protective against HIV infection among heterosexual sero-discordant couples (2, 3); women (4); men who have sex with men (MSM) (5); and injecting drug users (6). Evidence also shows that PrEP is safe to use by pregnant or lactating women (7–10). However, there are few surveys on knowledge and use of PrEP for HIV among pregnant and lactating women (11), and most of them have been conducted in South Africa. Data from the existing surveys showed low levels of awareness and knowledge about PrEP among pregnant and lactating women (12–14).

Eswatini has an HIV prevalence of 24.8% among adults 15 years and older, with a prevalence of 30.4% among women overall (15), and 35.4% among pregnant women aged 15–49 years (16). This is much higher than the prevalence among similarly-aged men of 18.7% (15). The annual HIV incidence rate among adults 15–49 years is 0.77% (0.20% among males and 1.45% among females) (15). With the high HIV prevalence and incidence rates, pregnant and post-natal women (PPW) in Eswatini are at substantial risk of HIV infection (11). Women who get infected with HIV during pregnancy or breastfeeding risk transmitting HIV to their infants (11). In 2019, the Eswatini Ministry of Health scaled-up the provision of PrEP as part of combination HIV prevention, with a particular focus on HIV-negative PPW, adolescent girls and young women (AGYW) aged 16–24 years, men aged 30–34 years, HIV-negative partners in sero-discordant sexual relationships, clients with sexually transmitted infections (STIs), and key populations (sex workers, men who have sex with men and transgender clients) (11). In Eswatini, clients are eligible for PrEP if at substantial risk after an assessment; age is 16 years and above; HIV test is negative on the day of PrEP initiation; there is no presence of symptoms indicating acute HIV infection (AHI) in combination with an exposure for HIV in the previous 14 days; willing to attended PrEP visits until 28 days after risk period; no contraindication to TDF + lamivudine (3TC); and bodyweight is 30 kilograms (kg) and above. PPW women are considered to be at substantial risk for HIV infection and are offered PrEP upon testing HIV-negative and having no contraindications for PrEP.

In Eswatini there is paucity of data on facilitators and barriers among PPW. Understanding the levels of knowledge, intention, use and potential gaps related to PrEP among PPW could help to identify opportunities for education and program implementation. The information could also be used to monitor the impact of social behavior change activities aiming to improve knowledge, attitudes and practices related to PrEP in antenatal and postpartum women. This study aimed to determine oral PrEP related levels of knowledge, attitudes, intention and practices PPW in Eswatini, and also to determine factors associated with use and intention to use PrEP among PPW.

## Materials and methods

### Study design

We conducted a cross-sectional survey among HIV- negative PPW between September 2021 and November 2021. All HIV-negative women receiving antenatal care (ANC) and postnatal care (PNC) services in the study sites were invited to participate in the study. Individual interviews were conducted using a structured questionnaire covering topics on socio-demographic characteristics, HIV risk behaviors, PrEP knowledge, PrEP access and sources of information, PrEP experiences (e.g., adherence, discontinuity, and side effects), intention to use PrEP and PrEP stigma. Only women who provided written informed consent were interviewed.

### Study sites

The study was conducted in 16 purposively selected PEPFAR-supported health facilities and regions (Hhohho and Shiselweni) in Eswatini which were offering oral PrEP to PPW. By November 2020 there were 55 health facilities providing PrEP to PPW in Hhohho and Shiselweni regions, of which 34 were in Hhohho and 21 were Shiselweni region. Among the health facilities, six were public health units (PHUs) and 49 were clinics. Public health units provide primary healthcare services and are the basis for outreach services in Eswatini while clinics only provide primary healthcare services. Since there was a small number of PHUs, all six PHUs were included in the study and five clinics were randomly selected from each region using a random number generator in Microsoft Excel. Nine sites were selected from Hhohho region and seven sites were from Shiselweni region. The study sites comprised of four sites located in urban areas and 12 sites located in rural areas.

### Sample size

The sample size calculation aimed to provide a sufficient sample size to estimate the proportion of PPW with knowledge about PrEP with ±3% precision (half width of 95% Wilson confidence intervals) or better. Since the proportion of PPW with knowledge about PrEP in Eswatini was unknown, the sample size calculation assumed that 50% PPW would have knowledge about PrEP. In addition, the sample size had to be large enough to allow a detection of significant differences of at least 10% with 80% power in knowledge and attitudes between current PrEP users and non-users, and also large enough to perform multivariate analysis to determine factors associated with PrEP use and intention to use PrEP. A sample size of at least 1,064 was required to be able to meet the study objectives. We also factored in 10% refusal and non-response rate. Probability proportional to size was used to select the number of PPW to be interviewed from each site.

### Study population and eligibility

The study population comprised of HIV-negative PPW seeking antenatal and post-natal care in the study sites. Women were eligible to participate in the study if they were accessing antenatal or post-natal services at the sites; were 18 years or older; were pregnant or reported to have delivered within 24 months; had a documented HIV-negative status; willing to provide consent to participate, and able to read and/or speak one of the study languages of English and SiSwati. Women were excluded if they had an illness that could prevent their participation, which included display of pain, inability to focus on the conversation or to talk or to sit throughout the interview.

### Participant recruitment and data collection procedures

Women were recruited within Maternal Child and Neonatal Health (MNCH) departments in the study sites with the support of healthcare workers (HCWs) who worked in the study sites. The HCWs informed potential study participants about the study after providing them with the required clinical services for the day. Interested women were referred to trained research assistants (RAs) who were stationed in the study sites. After obtaining written informed consent, RAs interviewed the women in a quiet space using a structured questionnaire designed in EpiInfo 7, entering the responses directly into the database on Wi-Fi enabled tablets. The questionnaire had built-in controls and checks to assure data accuracy and quality.

### Data collection instrument and definition of terms

A questionnaire was developed specifically for the study by adapting already validated questions from similar Knowledge, Attitude and Practices (KAP) surveys in multiple populations (12, 14, 17, 18). Adaptation included removing or rephrasing words and statements which did not apply to the study population. The interviewer-administered questionnaire collected data on socio-demographic characteristics, HIV risk behaviors, PrEP awareness and knowledge, PrEP access and sources of information, PrEP experiences (e.g., adherence, discontinuity, side effects), PrEP attitudes, PrEP motivation, PrEP self-efficacy, PrEP willingness, PrEP potential uptake (intention), and PrEP stigma. The questionnaire was translated from English to SiSwati, and participants had the interview in English or SiSwati depending on their preference.

**Socio-demographic characteristics and sexual behavior** questions were adapted from a PrEP demonstration survey conducted in Nigeria (17) and from a survey of knowledge and PrEP use among pregnant and breastfeeding women (PBFW) in South Africa (12). Socio-demographic variables included age, level of education, marital status, employment status, health related decision making and characteristics of male partners such as their age, HIV status, and employment. Sexual behavioral variables included number of sexual encounters, number of sexual partners, condom use, use of post exposure prophylaxis (PEP) for HIV, and testing and treatment for sexually transmitted illnesses (STIs).

Questions about **PrEP awareness, sources of information and places to access PrEP** were adapted from the survey of knowledge and PrEP use among PBFW in South Africa (12). Participants were asked if they have ever heard about PrEP, and where they have heard about PrEP. They were also asked where they would like to get information about PrEP and access PrEP pills in the future.

The questions about PrEP experience were adapted from the PrEP demonstration survey in Nigeria, the Durbar Mahila Samanwaya Committee (DMSC) case study and the Ashodaya Samithi Demo and Feasibility Project, and from the AIDS Clinical Trials Group (ACTG) Adherence Baseline Questionnaire (17). Participants were asked to self-report on the use of PrEP, experience of side effects, adherence to PrEP medication, PrEP disclosure and reasons for discontinuing PrEP for those who had discontinued PrEP.

For the study **PrEP users** were defined as women who were using PrEP at the time of the survey and **PrEP non users** were women who had never used PrEP and those who had previously used PrEP but had stopped taking PrEP at the time of the survey. Two questions were used to determine use of PrEP (1) Have you ever taken PrEP pills and (2) Are you currently taking PrEP pills. The response options were “Yes”, “No” and “Do not remember/ Refused to answer”. Only respondents who answered “Yes” were considered to have ever used or were currently using PrEP.

**Knowledge about PrEP**: was measured using four items as follows: (1) Consistent use of PrEP reduces HIV risk among HIV-negative individuals, (2) People using PrEP are recommended to continue using condoms, (3) Inconsistent use of PrEP decreases its effectiveness and (4) PrEP does not help prevent other STIs. The items were adapted from a survey of PrEP functional knowledge among MSM conducted in 2018 and were validated as part of scale on “Functional Knowledge of HIV Prevention Strategies” in the survey “Prioritizing U, 2015” (18). The response options were “True”, “False”, and “Do not know”. If a respondent answered “True” to ALL of the four items, then they were considered to have knowledge about PrEP otherwise considered not having knowledge about PrEP.

Before introducing respondents to PrEP scales, a summary of the meaning of PrEP was orally presented as follows. “*There is a new way to prevent HIV infection for people who may be exposed to the virus. It is called Pre-Exposure Prophylaxis or PrEP. It involves an HIV-negative person taking a pill daily, on an ongoing basis (starting before an exposure and continuing after for as long as the person is at risk) to reduce their risk of HIV infection. Research suggests that PrEP is generally safe and is highly effective (over 90%) in preventing HIV infection if taken every day. It is much less effective if not taken every day and does not protect against other sexually transmitted infections. Taking PrEP would require a visit to a doctor every three months in order to be tested for HIV, STIs and side effects* (19).”

**PrEP Attitudes**: was measured using a 5-items scale which sought to assess the participant's beliefs around PrEP's safety and effectiveness at preventing HIV (Supplementary Table S1). The items were taken from the article on applying the Information-Motivation-Behavioral Skills Model to understand PrEP intentions and use among MSM (20).The response options were 1 = Strongly disagree, 2 = Disagree, 3 = Neutral, 4 = Agree, 5 = Strongly agree. The scale was constructed by summing the item scores and dividing by the number of items. The scale score range was 1–5, and a higher scale score indicated a higher level of positive PrEP attitudes. Using Factor Analysis, the scale was reliable to measure attitudes towards PrEP with Cronbach's Alpha (α) = 0.7.

**PrEP Self-efficacy**: was measured using a 5-items scale (α = 0.8) which sought to assess the participant's perceived ability to take PrEP consistently and as required (19) (Supplementary Table S1). The response options were: 1 = Not at all confident, 2 = Slightly confident, 3 = Somewhat confident, 4 = Fairly confident, 5 = Completely confident. The scale was constructed by summing the item scores and dividing by the number of items. The scale score range was 1–5, and a higher scale score indicated a higher level of self-efficacy (belief in self to use PrEP correctly).

**PrEP Motivation**: was measured using a 6-items scale (α = 0.6) which sought to assess circumstances under which participants would take or not take PrEP (19). These included assessing if clients would want to take PrEP if they knew about PrEP side effects, if they had to disclose to their sexual partners about taking PrEP, if they knew someone taking PrEP, if they had social support and if they trusted the efficacy of PrEP to prevent HIV transmission (Supplementary Table S1). The response options were: 1 = Strongly disagree, 2 = Disagree, 3 = Neutral, 4 = Agree, 5 = Strongly agree. The scale was constructed by summing the item scores and dividing by the number of items. The scale score range was 1–5, and a higher scale score indicated a higher level of motivation to use PrEP.

**PrEP Stigma**: was measured using a 13-items scale (α = 0.8) which covered fear of being perceived as promiscuous and fear of being shunned or rejected within social circles (Supplementary Table S1). The items were adapted from “The Pre-Exposure Prophylaxis (PrEP) Stigma Scale” (14, 21). The response options were: 1 = Strongly agree, 2 = Agree, 3 = Neutral, 4 = Disagree and 5 = Strongly Disagree. The scale was constructed by summing the item scores and dividing by the number of items. The scale score range was 1–5, a lower score indicated a higher level of stigma about PrEP.

**PrEP Intention:** was measured using three items as follows: (1) During the next three months, I will talk to a health care provider about PrEP; (2) During the next three months, I will seek out more information about PrEP and (3) During the next three months, I will get a prescription for PrEP (20). The response options were: 1 = No, definitely not; 2 = No, probably not; 3 = Yes, probably; 4 = Yes, definitely. A women was considered to have intention to use PrEP if they responded with either option “3 = Yes, probably” or option “4 = Yes, definitely” across all the three statements.

**PrEP Willingness**: was measured using a 6-items scale (α = 0.8) adapted from a study on willingness to take PrEP for HIV prevention among Thai MSM (22) (Supplementary Table S1). The items assessed participants' willingness to take PrEP if available, even if they would still have to use condoms, if it could cause temporary mild side effects, if they had to pay for it and if they would still need to test regularly for HIV. The response options were: 1 = No, definitely not; 2 = No, probably not; 3 = Yes, probably; 4 = Yes, definitely. The scale was constructed by summing the item scores and dividing by the number of items. The scale score range was 1–4, a higher score indicated a higher level of willingness to use PrEP.

### Data analysis

Data analysis was performed using the Statistical Package for Social Sciences (SPSS) version 26.0. Categorical variables were summarized using frequencies and percentages of participants. Continuous variables were summarized using means and standard deviations or medians and interquartile ranges, as appropriate. Factor analysis was used to confirm reliability among the scale items. Scales were considered reliable if the Cronbach's alpha was 0.5 and above. Pearson's chi-squared test was used to measure the association among categorical variables, and a rank-sum test was used to measure association between continuous variables. The precision around PrEP awareness, knowledge, use and intention estimates was assessed by 95% confidence intervals. Additionally, we used multivariate logistic regression to identify predictors of PrEP use and intention to use PrEP. Odds ratios and their respective 95% confidence intervals were used to quantify the effects. Variables for the multivariate model, were initially compiled informed by background knowledge, and these included region, age, education, marital status, decision making about health-related issues, HIV testing, male partner's characteristics, sexual risk behavior, self-efficacy, social support, perceived benefits and barriers of PrEP and stigma association with taking PrEP (14, 23–25). The potential factors were subsequently screened using simple (i.e., univariate) logistic regression and included in the multivariate model if the association with the respective dependent variable had a *p*-value of 0.05 or lower. Missing cases were excluded in a listwise fashion.

### Ethical considerations

The protocol was implemented with human subject oversight provided by the Eswatini Health and Human Research Review Board (EHHRRB) (IRB: 00011253) in Eswatini, and the Advarra IRB (IORG0000468) in the United States of America. In addition, administrative approvals were obtained from the Eswatini National AIDS Program; Regional Health Management Teams, and senior management teams at the study sites. No monetary incentives were provided to the women for being part of this study. To enhance confidentiality, study participants were assigned unique study identification numbers to identify and link study records.

## Results

**Figure 1:**
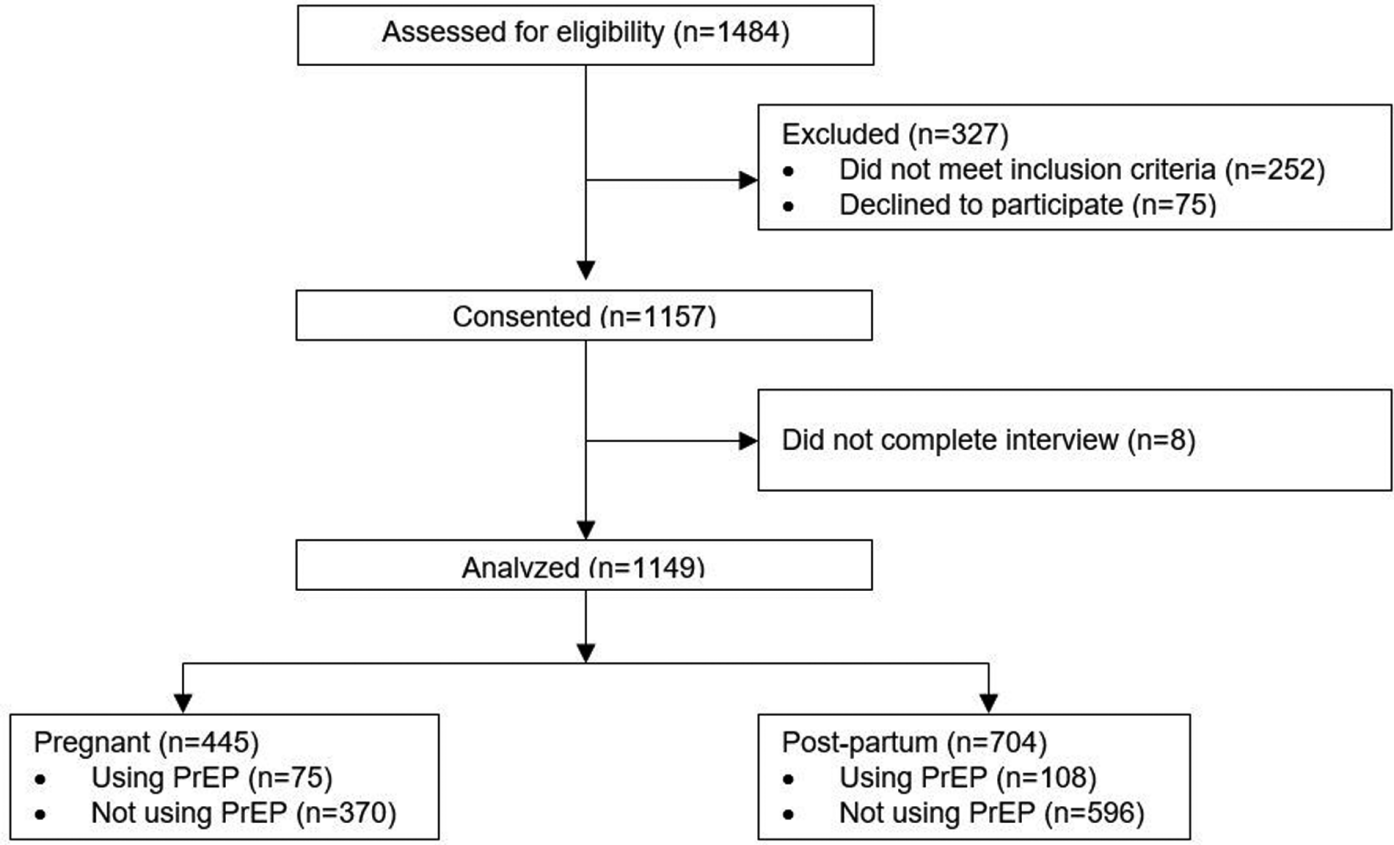
Flow of study screening and enrollment.

A total of 1,484 PPW were referred to the Research Assistants by Healthcare Workers (HCWs) in study sites, and 252 (17.0%) were not eligible to participate in the study, 1,157 (78.0%) consented to participate in the study and 75 (5.0%) did not consent. Of the 75 women who did not consent to participate, 23 (30.7%) refused (did not want/ not interested/not comfortable), 46 (61.3%) did not have enough time to sit through the interview/in a hurry to leave health facility, 6 (8.0%) had their children crying endlessly. Among the 1,157 PPW who consented to participate in study, 1,149 completed the interview. Figure 1 presents the flow of study participant screening and enrollment.

### Characteristics of respondents

A total of 1,149 PPW women were interviewed for the study: 704 (61.3%) women were post-partum and 445 (38.7%) were pregnant (Table 1). The median age was 25 years [Interquartile Range (IQR) = 21–30 years], with 46.4% (533) 18–24 years old. Most women (*n* = 902, 78.8%) said that they are the ones who usually make decisions regarding their health, 100 (8.7%) had their male partners making the decisions, 111 (9.7%) their parents. Nearly two-thirds, 748 (65.3%) had HIV-negative male partners, 83 (7.2%) had HIV-positive male partners and 318 (27.7%) did not know the HIV status of their male partners. Of the women who reported that their male partners were HIV-positive, 70 (84.3%) reported that the HIV-positive male partners were on antiretroviral therapy (ART).

**Table 1:** Characteristics of study participants.

Characteristic	Total (*N* = 1,149)	Pregnant (*N* = 445)	Postpartum (*N* = 704)
	*n* (%)	*n* (%)	*n* (%)
Age (years)			
Median (IQR)	25 (21–30)	24 (21–29)	26 (22–31)
Categories			
18–24	533 (46.4)	227 (51.0)	306 (43.5)
25–29	304 (26.4)	107 (24.0)	197 (28.0)
30–34	203 (17.7)	81 (18.2)	122 (17.3)
36–39	84 (7.3)	24 (5.4)	60 (8.5)
40+	25 (2.2)	6 (1.4)	19 (2.7)
Level of education			
None	9 (0.8)	4 (0.9)	5 (0.7)
Primary (first 7 years of school)	156 (13.6)	54 (12.2)	102 (14.5)
Secondary (1–3 classes post primary)	337 (29.4)	126 (28.4)	211 (30.0)
High school (4–5 classes post primary level)	470 (40.9)	181 (40.8)	289 (41.1)
Tertiary (Post high school)	176 (15.3)	79 (17.8)	97 (13.8)
Missing	1	1	0
Marital status			
Married	396 (34.5)	154 (34.6)	242 (34.5)
Cohabiting	129 (11.2)	50 (11.2)	79 (11.3)
Not married	622 (54.2)	241 (54.2)	379 (54.3)
Missing	2	0	2
Employment status			
Unemployed	759 (66.1)	275 (61.8)	484 (68.8)
Student	65 (5.7)	37 (8.3)	28 (4.0)
Employed	325 (28.3)	133 (29.9)	192 (27.3)
Person who makes decisions regarding health			
Myself	902 (78.8)	351 (79.1)	551 (78.6)
My partner	100 (8.7)	29 (6.5)	71 (10.1)
Parent/s	111 (9.7)	51 (11.5)	60 (8.6)
Someone else	32 (2.9)	13 (2.9)	19 (2.7)
Missing	4	1	3
Period tested for HIV			
Before the pregnancy	140 (12.2)	56 (12.6)	84 (12.0)
During pregnancy	466 (40.7)	366 (82.4)	100 (14.2)
After delivery	540 (47.1)	22 (5.0)	518 (73.8)
Missing	3	1	2
Region			
Hhohho	850 (74.0)	323 (72.6)	527 (74.9)
Shiselweni	299 (26.0)	122 (27.4)	177 (25.1)
Male partners’ age (years)			
Median (IQR)	31 (26–36)	30 (25–35)	32 (27–38)
Categories			
18–24 years	169 (14.7)	82 (18.4)	87 (12.4)
25–29 years	277 (24.1)	110 (24.7)	167 (23.7)
30–34 years	267 (23.2)	118 (26.5)	149 (21.2)
36–39 years	183 (15.9)	63 (14.2)	120 (17.0)
40 years and above	167 (14.5)	44 (9.9)	123 (17.5)
Unknown	86 (7.5)	28 (6.3)	58 (8.2)
Age gap between participants and their male partners			
1–5 years	593 (51.6)	239 (53.7)	354 (50.3)
6–10 years	346 (30.1)	141 (31.7)	205 (29.1)
11 years and above	124 (10.8)	37 (8.3)	87 (12.4)
Age gap unknown	86 (7.5)	28 (6.3)	58 (8.2)
Occupation of male partner			
Not working	189 (16.4)	58 (13.0)	131 (18.6)
Student	41 (3.6)	21 (4.7)	20 (2.8)
Working	914 (79.5)	364 (81.8)	550 (78.1)
Missing	5	2	3
HIV status of male partner			
HIV-negative	748 (65.1)	300 (67.4)	448 (63.6)
HIV-positive	83 (7.2)	28 (6.3)	55 (7.8)
Do not know	318 (27.7)	117 (26.3)	201 (28.6)
HIV-positive male partner on ART			
No	5 (6.0)	4 (14.3)	1 (1.8)
Yes	70 (84.3)	20 (71.4)	50 (90.9)
Do not know	8 (9.6)	4 (14.3)	4 (7.3)
HIV-negative male partner on PrEP			
No	698 (93.1)	283 (94.3)	413 (92.2)
Yes	7 (0.9)	0 (0.0)	7 (1.6)
Do not know	45 (6.0)	17 (5.7)	28 (6.2)

### Sexual behavior of respondents

Of the 1,149 women interviewed, 353 (30.8%) reported to have used a condom in their last sex encounter while 154 (21.8%) reported to have used a condom every time they had sex in the last month and 415 (58.6%) had never used a condom during sex in the last month (Table 2). About 370 (32.3%) had tested for a sexually transmitted infection (STI) in the past 6 months; 55 (4.8%) had been treated for a Sexually Transmitted Infection (STI) in the past 3 months; 28 (2.5%) had engaged in anal sex in the past 3 months; and 127 (11.1%) had taken post-exposure prophylaxis (PEP) following a potential exposure to HIV in the past six months.

**Table 2:** Sexual behavior.

Characteristic	Total (*N* = 1,149)	Pregnant (*N* = 445)	Post-partum (*N* = 704)
	*n* (%)	*n* (%)	*n* (%)
Had sexual intercourse in the last month			
No	404 (35.2)	93 (20.9)	311 (44.2)
Yes	745 (64.8)	352 (79.1)	393 (55.8)
Number of times a condom was used with partner in the past month			
Never	415 (58.6)	230 (67.1)	185 (50.7)
Sometimes	117 (16.5)	59 (17.2)	58 (15.9)
Most of the time	22 (3.1)	5 (1.5)	17 (4.7)
Every time	154 (21.8)	49 (14.3)	105 (28.8)
Missing	37	9	28
Condom use during the last sex encounter			
No	793 (69.2)	347 (78.0)	446 (63.6)
Yes	353 (30.8)	98 (22.0)	255 (36.4)
Tested for a sexually transmitted infection (STI) In the past 6 months			
No	769 (67.2)	261 (59.5)	508 (72.6)
Yes	370 (32.3)	178 (40.5)	192 (27.4)
Treated for an STI in the past 3 months			
No	1,087 (95.1)	425 (96.4)	662 (94.4)
Yes	55 (4.8)	16 (3.6)	39 (5.6)
Engaged in anal sex in the past 3 months			
No	1,088 (97.50	425 (98.2)	663 (97.1)
Yes	28 (2.5)	8 (1.8)	20 (2.9)
Taken post-exposure prophylaxis (PEP) following a potential exposure to HIV in the past six months			
No	1,019 (88.7)	392 (88.5)	627 (89.2)
Yes	127 (11.1)	51 (11.5)	76 (10.8)

### PrEP awareness and knowledge

Over 80% (*n* = 930, 80.7%) of the women had ever heard about oral PrEP for HIV prevention, while 219 (19.3%) had never heard about PrEP. Most women, 706 (76.2%) heard about PrEP at a clinic or hospital, followed by 150 (16.2%) who had heard about PrEP at a community or outreach event (Supplementary Table S2). Similarly, the most preferred source of information about PrEP among the women was a clinic or hospital (*n* = 1,039, 90.4%), followed by the community or outreach event (*n* = 151, 13.1%). The clinic or hospital was also preferred by a majority (*n* = 1,112, 96.8%) of the women for accessing PrEP pills, followed by the community or outreach event (*n* = 101, 8.8%). The proportion of PPW who knew all four facts (1) Consistent use of PrEP reduces HIV risk among HIV-negative individuals, (2) People using PrEP are recommended to continue using condoms, (3) Inconsistent use of PrEP decreases its effectiveness and (4) PrEP does not help prevent other STIs) about PrEP was 635 (55.3%) (95 CI: 52.3, 58.2).

### PrEP attitudes and stigma

The women had positive attitudes towards PrEP. A majority of the women, 1,032 (89.8%) either agreed or strongly agreed that taking PrEP is safe; 974 (84.8%) agreed that PrEP is effective for preventing HIV; and 859 (74.8%) felt that it was not going to be difficult to adhere to PrEP every day (Table 3). Regarding stigma, a majority of the women either agreed or strongly agreed that if they were to use PrEP people would think they have HIV (*n* = 759, 66.0%), and that people would think that they have sex with a lot of different people (*n* = 658, 57.3%) (Table 3). Nearly half of the women (*n* = 511, 44.5%) felt that if they brought-up the subject of PrEP with their partners, then their partners would think that they are having risky sex with other people.

**Table 3:** PrEP attitudes and stigma among respondents.

Statement	Agree/strongly agree (*N* = 1,149)
	*n* (%)
Attitudes	
Taking PrEP is safe	1,032 (89.8)
PrEP is effective at preventing HIV	974 (84.8)
The government makes certain that drugs like PrEP are safe	956 (83.2)
People who take PrEP are responsible	935 (81.4)
It would be no trouble to take PrEP every day	859 (74.8)
Stigma-fear of being perceived as promiscuous	
If I were to use PrEP, people would think that I have HIV	759 (66.0)
If I were to use PrEP, people would think that I have sex with a lot of different people	658 (57.3)
If I were to use PrEP, people would think that I like having strange types of sex	612 (53.3)
If I were to bring up the subject of using PrEP with my partner, he would think that I am having risky sex with other people	511 (44.5)
Stigma-fear of being shunned	
My friends would think less of me if they found out I was using PrEP	449 (39.1)
People would feel uncomfortable with me if they found out that I used PrEP	359 (31.2)
People would avoid me if they found out that I used PrEP	289 (25.2)
If I used PrEP, I would worry that people would tell others that I am using PrEP	342 (29.8)
My family would think less of me if they found out I was using PrEP	291 (25.3)
I would worry about telling people that I take a medicine like PrEP for my health's sake	278 (24.2)
If I were going to use PrEP, I would feel a need to hide that from other people	266 (23.2)

### Use and experiences with using PrEP

Among all the 1,149 PPW interviewed for the study, the number of PPW who have ever used PrEP was 285 (24.8%), and the number of PPW who were currently using PrEP was 183 (15.9%) (Supplementary Table S3). Among the 285 women who have ever used PrEP, 102 (35.8%) had stopped using PrEP. Seventy eight (76.5%) PPW had stopped taking PrEP within the past 12 months, and 12 (13.3%) in more than a year. Thirty-five (35.2%) PPW had stopped taking PrEP due to side effects; and 59 (64.8%) stopped due to other reasons including unavailability of PrEP pills (*n* = 15, 27.8%), perceived lack of HIV acquisition risk (*n* = 7, 13.0%) and partners/husbands' refusal (*n* = 6, 11.1%). When asked if they would like to re-start taking PrEP, 62 (60.8%) wanted to start taking PrEP again, among which 23 (42.6%) wanted to protect themselves from getting infected with HIV, 8 (14.8%) because they did not trust their partners, and the remaining due to a variety of reasons. Of the 183 PPW who were still on PrEP during the survey; 63 (34.4%) reported to have experienced side effects as a result of taking PrEP; 142 (77.6%) had disclosed to their male partners about taking PrEP; 91 (49.7%) had disclosed to their parents; and 54 (29.5%) had disclosed to other family members (Supplementary Table S3). When asked if they would want to continue taking PrEP in the next month, 174 (95.6%) wanted to continue using PrEP for the next month and 8 (4.4%) did not.

### Factors associated with PrEP use

Using multivariate logistic regression, we determined factors associated with the use of PrEP. We first used univariate logistic regression to measure association of potential factors to “PrEP use” and only included in the final multivariate model factors that were significant with a *p* = value of 0–05 or lower. PrEP use, comparing women on PrEP against women not on PrEP, was associated with HIV status of male partner, PrEP attitudes, and self-efficacy (Table 4). Women with an HIV-positive male partner were more likely to use PrEP compared to women with a HIV-negative partner [adjusted odds ratio [aOR] = 7.8, 95% confidence interval (CI) (3.5, 17.0)]. PPW with positive PrEP attitudes [aOR = 1.6, 95% CI (1.0, 2.4)]; and high self-efficacy about taking PrEP correctly [aOR = 1.5, 95% CI (1.1, 2.0)] were more likely to use PrEP.

**Table 4:** Factors associated with PrEP use among pregnant and post-partum women.

Factors	Un-adjusted odds ratio (95% CI)	*p*-value	Adjusted odds ratio (95%CI)	*p*-value
Region				
Shiselweni	REF		REF	
Hhohho	2.9 (1.8, 4.7)	<0.001	1.5 (0.8, 2.6)	0.207
Pregnant or post-partum				
Post-partum	REF			
Pregnant	1.1 (0.8,1.5)	0.495		
Age groups		***0.270***	** **	** **
40 + years	REF			
18–24 years	1.2 (0.4,3.4)	0.798		
25–29 years	0.8 (0.3,2.4)	0.652		
30–34 years	0.8 (0.3,2.6)	0.765		
36–39 years	1.2 (0.4,4.1)	0.730		
Level of education		***0.265***	** **	** **
Tertiary (post high school education)	REF			
No schooling or primary (first 7 years of school)	1.7 (0.9,3.2)	0.078		
Secondary (1–3 classes post primary level)	1.4 (0.8,2.5)	0.210		
High school (4–5 classes post primary level)	1.6 (1.0,2.7)	0.069		
Marital status		***0.152***	** **	** **
Not married	REF			
Married	1.1 (0.8,1.5)	0.628		
Cohabiting	1.6 (1.0,2.6)	0.053		
What is your occupation?		***0.565***	** **	** **
Employed	REF			
Unemployed	1.2 (0.8,1.8)	0.289		
Student	1.1 (0.5,2.3)	0.796		
Age gap between male partner and participant		***0.293***	** **	** **
Age gap unknown	REF			
1–5 years	0.7 (0.4,1.2)	0.190		
6–10 years	0.8 (0.4,1.4)	0.428		
11 years and above	1.0 (0.5,2.0)	0.944		
Decision maker regarding respondent's health		***0.159***	** **	** **
Someone else	REF			
Myself	1.1 (0.4,2.9)	0.843		
My partner	0.8 (0.3,2.5)	0.707		
Parent/s	0.5 (0.2,1.7)	0.288		
Male partner's HIV status		***<0.001***	** **	**<0**.**001**
HIV negative	REF		REF	
HIV positive	13.5 (7.5, 24.4)	<0.001	7.8 (3.5,17.0)	<0.001
HIV status unknown	1.8 (1.1, 2.8)	0.001	1.6 (0.9,2.8)	0.141
Condom use during the last sex encounter				
No	REF			
Yes	1.0 (0.7,1.4)	0.912		
Tested for an STI in last 6 months				
No	REF		REF	
Yes	0.5 (0.4, 0.7)	<0.001	0.7 (0.4,1.1)	0.086
Treated for an STI in the past 3 months				
No	REF			
Yes	0.8 (0.4,1.5)	0.411		
Engaged in anal sex in the last 3 months				
No	REF			
Yes	0.6 (0.2,1.3)	0.181		
Taken PEP in last 6 months				
No	REF		REF	
Yes	0.5 (0.4, 0.7)	<0.001	0.1 (0.0, 0.1)	<0.001
PrEP perceptions				
Attitudes	2.5 (1.8, 3.3)	<0.001	1.6 (1.0, 2.4)	0.042
Motivation	1.4 (1.1, 1.8)	0.004	1.3 (0.9,1.8)	0.152
Self-efficacy	1.8 (1.5, 2.3)	<0.001	1.5 (1.1,2.0)	0.005
Stigma	1.5 (1.2,1.9)	<0.001	1.3 (1.0,1.7)	0.087

The bold italics indicate that the *p*-values for the associated of the covariates (as a whole) with the dependent variable.

### Intention to use PrEP

Regarding intention to use PrEP, among 864 PPW who had never used PrEP, 65.3% intended to use PrEP in the future and 34.1% did not. The intention to use PrEP, comparing women who intended to use PrEP against women who did not intend to use PrEP, was associated with level of education, PrEP awareness, willingness to use PrEP, and self-efficacy (Table 5). PPW who had attained high school education [aOR = 1.7, 95% CI (1.1, 2.8)], secondary education [aOR = 2.2, 95% CI (1.3, 3.9)] and had primary education or no schooling [aOR = 2.0, 95% CI (1.1, 3.9)] are more likely to intend to use PrEP compared to those who have attained tertiary education. PPW who have ever heard about PrEP [aOR = 1.7, 95% CI (1.1, 2.6)]; high willingness to use PrEP [aOR = 3.1, 95% CI (2.3, 4.1)]; and with high self-efficacy (believe that they are capable of taking PrEP as required) [aOR = 1.6, 95% CI (1.3, 1.9)] are more likely to intend to use PrEP.

**Table 5:** Factors associated with intention to use PrEP among pregnant and post-partum women.

Factors	Un-adjusted odds ratio (95% CI)	*p*-value	Adjusted odds ratio (95%CI)	*p*-value
Region				
Shiselweni	REF			
Hhohho	1.1 (0.8,1.6)		0.376	
Pregnant or post-partum				
Post-partum	REF			
Pregnant	1.1 (0.8,1.4)	0.644		
Age groups		***0.672***	** **	** **
40 + years	REF			
18–24 years	1.3 (0.5,3.6)		0.632	
25–29 years	1.0 (0.3,2.8)		0.990	
30–34 years	1.2 (0.4,3.3)		0.789	
36–39 years	1.1 (0.4,3.6)		0.819	
Highest level of education attained		***0.001***	*** ***	***0***.***022***
Tertiary (post high school education)	REF		REF	
No schooling or primary (first 7 years of school)	2.5 (1.5, 4.3)	<0.001	2.0 (1.1, 3.9)	0.028
Secondary (1–3 classes post primary level)	2.2 (1.5, 3.4)	<0.001	2.2 (1.3,3.9)	0.003
High school (4–5 classes post primary level)	1.9 (1.3,2.8)	0.002	1.7 (1.1,2.8)	0.025
Marital status		***0.104***	** **	** **
Not married	REF			
Married	0.9 (0.7,1.2)	0.436		
Cohabiting	1.6 (0.9,2.7)	0.081		
What is your occupation?		***0.580***	** **	** **
Employed	REF			
Unemployed	1.2 (0.9,1.6)	0.300		
Student	1.2 (0.6,2.2)	0.651		
Decision maker regarding respondent's health		***0.024***	*** ***	***0***.***901***
Someone else	REF		REF	
Myself	1.1 (0.4,2.5)	0.886	1.0 (0.3,3.0)	0.981
My partner	1.3 (0.5,3.4)	0.591	0.9 (0.2,3.0)	0.846
Parent/s	2.4 (0.9,6.4)	0.074	1.2 (0.4,4.2)	0.742
Age gap between male partner and participant		***0.459***	** **	** **
Age gap unknown	REF			
1–5 years	1.5 (0.9,2.6)	0.114		
6–10 years	1.5 (0.8,2.6)	0.188		
11 years and above	1.3 (0.7,2.6)	0.380		
Male partner's HIV status		***0.112***	** **	** **
HIV negative	REF			
HIV positive	0.8 (0.6,1.1)	0.259		
HIV status unknown	2.3 (0.8,7.0)	0.134		
Condom use during the last sex encounter				
No	REF			
Yes	1.0 (0.7,1.4)	0.998		
Tested for an STI in last 6 months				
No	REF			
Yes	1.2 (0.9,1.6)	0.256		
Treated for an STI in the past 3 months				
No	REF			
Yes	1.4 (0.7,2.8)	0.320		
Engaged in anal sex in the last 3 months				
No	REF			
Yes	1.0 (0.4,2.7)	0.918		
Taken PEP in last 6 months				
No	REF			
Yes	1.2 (0.4,3.7)	0.739		
Ever heard about PrEP (PrEP awareness)				
No	REF		REF	
Yes	1.5 (1.1, 2.1)	0.015	1.7 (1.1,2.6)	0.013
PrEP Perceptions				
Willingness	4.3 (3.4, 5.5)	<0.001	3.1 (2.3,4.1)	<0.001
Attitudes	2.3 (1.9,2.9)	<0.001	1.2 (0.9,1.5)	0.306
Motivation	2.3 (1.9,2.8)	<0.001	1.2 (1.0,1.6)	0.094
Self-Efficacy	2.2 (1.9,2.5)	<0.001	1.6 (1.3,1.9)	<0.001
Stigma	1.5 (1.3,1.8)	<0.001	1.1 (0.9,1.4)	0.298

The bold italics indicate that the *p*-values for the associated of the covariates (as a whole) with the dependent variable.

## Discussion

This study identified that a majority of the PPW had ever heard of PrEP, however, about 19% did not know anything about it. Additionally, only about half of PPW possessed the correct knowledge about PrEP. This is an indication of gaps in health education, particularly within health facilities, which is where the women were recruited for the study. Whilst this study shows slightly higher proportions of PPW aware and also having correct knowledge about PrEP, several studies in similar populations in different settings in Sub-Saharan Africa and the United States of America have reported relatively low awareness and knowledge about PrEP (12, 26–32). In South Africa, in a study among pregnant women from Cape Town knowledge about PrEP was only 33% (12) and in a study among young pregnant women aged 18–24 years old in KwaZulu Natal, none of the women had ever heard about PrEP before the survey (32). In Zambia, knowledge about PrEP among pregnant and breastfeeding was only 36% (28). In the United States approximately two thirds of pregnant women had never heard of PrEP before participating in the study (26). Of note is that even if the women were aware about PrEP, a majority tended to have incorrect knowledge about PrEP as an option for HIV prevention and also tended to have concerns about potential effects to their babies during pregnancy or breast feeding (27, 31).

Limited PrEP awareness and knowledge among the PPW is concerning because if women possess little or no knowledge on PrEP, then they are less likely to utilize the service even if it is offered at the healthcare facilities. From the studies it emerged clearly that high acceptability of PrEP is associated with knowledge about its efficacy in preventing the acquisition of HIV, and once PrEP was explained to the women, most of them reported positive attitudes towards PrEP and an interest to initiate PrEP (27, 29, 30, 32). PrEP programs targeting women at ANC or PNC need to develop appropriate interventions to increase health education on HIV PrEP among PPW both within health facilities and communities. Health education should aim to increase accurate PrEP knowledge and also motivate PPW to use PrEP as PPW are considered to be at high-risk of acquiring HIV.

In this study, the most cited source of PrEP information for women was the health facility. This finding indicates possible locations for health education interventions which could be implemented when the clients come for other health services. It could also be an indication of missed opportunities for health education outside the health facility environment. Extending PrEP promotion to the community can help to reach populations who do not regularly attend health facilities (34). Other studies have reported similar findings where health facilities and healthcare workers are the most cited sources of PrEP information (12, 28, 30, 31, 34). In this regard, HCWs play a critical role in delivering PrEP in antenatal and postpartum care to PPW. However, studies from South Africa and France showed that less than half of HCWs knew about PrEP, described inaccurate PrEP knowledge regarding effectiveness, and lacked clinical detail (35–38). The limited PrEP knowledge among HCWs will hinder their ability to educate patients correctly about PrEP and the confidence to prescribe PrEP There is therefore a need to address this gap by providing trainings to HCWs on information about PrEP safety, efficacy, and how to prescribe it to pregnant and breastfeeding women, and other population groups.

The study also revealed that there was stigma attached to PrEP use. Most women agreed that if they were to use PrEP, people would think they have HIV, have sex with a lot of different people and/or like having strange types of sex. The PPW also agreed that their partners would think they were having risky sex with other people. A similar study conducted in Uganda, South Africa and Zimbabwe, mentioned that participants would refrain from taking PrEP because of its association with antiretroviral therapy and HIV related stigma (39). Likewise, findings from Malawi and Zambia showed that PrEP stigma was linked to being perceived as promiscuous and being on ART due to the appearance of PrEP packaging (29). This stigma associated with PrEP leads to challenges in PrEP initiation, retention, and adherence (14, 30, 34, 40–43). Interventions to address stigma and public education on HIV/AIDS prevention should address the social and cultural norms that undermine PrEP's optimal use (44, 46). HIV prevention programs should also consider introducing long-lasting injectable PrEP, as it comes with benefits of administration only once every two months and invisibility as no pills are required to be carried and taken by the individual (44–46).

Despite WHO's recommendation to offer PrEP to all population groups at substantial risk of HIV infection, the uptake is persistently low (11). In this study, only a quarter of PPW women have ever used PrEP and an even lower percentage were on PrEP at the time of the study. On the other hand, a large proportion of the women engaged in sex with men who were HIV-positive or had unknown HIV status, and many of the women's sexual encounters did not involve the use of condoms. This low coverage of PrEP among the women engaged in unprotected sex is worrying and may explain the continued high incidence of HIV in Eswatini. The factors associated with PrEP use identified by this study included having a known HIV-positive male partner, a male partner with unknown HIV status, positive attitudes towards PrEP, high self-efficacy, having tested for an STI in the last 6 months, and having taken PEP in the last 6 months. This finding aligns with previous studies conducted in Zambia and Kenya among PPW where factors associated with PrEP use were: being a sero-different couple, having a partner of unknown HIV status, having a positive attitude towards PrEP (47), and a reactive syphilis test result (7). Some studies identified other pertinent factors for PrEP use such as: engaging in sex without a condom in the past six months, having experienced intimate partner violence (7, 29), the desire to safely conceive a child (23), being a drug injector, being homeless (48) and rape (29). Worth noting is that most factors associated with PrEP use are factors know to be associated with the risk of HIV acquisition. Literature has shown that understanding the risk of HIV infection strengthens the desire to seek information about PrEP (12) and that women with perceived risk for HIV acquisition had high interest to use PrEP (49). For this reason, it is imperative for HCW to be aware of risk factors for HIV acquisition in order to provide the opportunity to discuss expanded HIV prevention options with women who are at risk of HIV exposure.

Among PPW using PrEP in this study, more than a quarter experienced various side effects, with dizziness and headaches being the most reported. Consequently, 36% of these women stopped taking PrEP due to the side effects. This finding resonates with already existing literature on PrEP where side effects were stated as some of the reasons for stopping PrEP (50, 51). However, studies from Zimbabwe and Mozambique had contradictory findings where the experience of side effects was not perceived as a major reason for discontinuing the use of PrEP, and in such cases women developed coping strategies of dealing with side effects (23, 52). It is critical for HCWs to provide information about PrEP side effects to PPW during initiation and follow up, so that they cope with side effects without stopping PrEP.

Our study found other reasons for PrEP discontinuation including; unavailability of PrEP and being stopped by partner or husband. Consistent findings from regional studies reported PrEP stock-outs and needing partner or husband approval to take PrEP as barriers to PrEP uptake, adherence and retention (23, 39, 47). The lack of autonomy among women to make decisions concerning their health may present a barrier to PrEP uptake. Thus, male involvement in promoting PrEP uptake beyond healthcare spaces including the community and key leaders such as traditional leaders, religious leaders, political leaders and employers could increase the use of PrEP in this target population (56) Also, political will is key in developing interventions and policy reviews to address challenges contributing to PrEP drugs stock-outs.

Some reasons for stopping PrEP unique to other studies were: changes in partner relationships and doubting safety of PrEP in pregnancy (41), changing risk perception, lack of social support, PrEP stigma, pill fatigue, and loss of interest (43, 51). These findings suggest that appropriate education and messaging about PrEP use, effectiveness, and side effects to communities might improve PrEP uptake and persistence. In so doing, partners and family members would also be enlightened about the importance of PrEP and supporting PrEP users. Also, conceptualizing PrEP as an intervention that can be paused and later restarted based on HIV risk may help ease the pill burden (54).

From this study, among women who never used PrEP, 65%, intended to use PrEP in the future. The odds of intention to use PrEP were high among women with low levels of education, awareness of PrEP, high self-efficacy and high willingness to use PrEP. These findings are similar to other studies where women with awareness of PrEP and high self-efficacy showed increased willingness to use PrEP (28, 55). On the contrary, Scott et al. in a research among PPW found that self-efficacy was not associated with PrEP uptake intention (56). Further, positive attitudes, subjective norms (support or approval from significant other), maternal status and breastfeeding were other factors associated with PrEP use intention reported from existing evidence (28, 55). To increase intention to use PrEP, it is imperative to sensitize women on risk factors for HIV infection, to be empowered with knowledge and make informed choices about using PrEP.

### Strengths and limitations

The main strength of this study is that it includes a large sample size of PPW from 16 health facilities. The results reported in this study are speciﬁc to PPW, providing important insights to inform scale-up of PrEP services to PPW. However, the study also has some limitations. The main limitation of this study is that it only sampled from the population of those already accessing the health care facility, therefore it may not represent the perspective of those who do not access health care facilities. Additionally, the study relies on self-reported information about PrEP during the interviews which may be biased.

## Conclusion

The study showed that there are gaps in PrEP awareness and knowledge and that PrEP uptake among PPW was low. While PPW generally believed that PrEP is safe and effective to prevent HIV, they were concerned about possible side effects and encountering negative experiences if they were to disclose about taking PrEP to their sex partners. There is a need to strengthen health education about PrEP for PPW. This should include improving the integration of PrEP counselling into existing clinic visits at ANC and PNC and offering clients with options for HIV prevention. The program can also improve efforts to identify and educate sero-discordant couples; continue implementing couples' HIV testing to ensure that the women have knowledge of their risk; and identify outreach strategies to be implemented at community level to reduce stigma and misinformation around PrEP. These strategies can reach women who may become pregnant (intentionally or otherwise), and possibly increase acceptability of PrEP early in the antenatal course. In addition, the study suggests that many women are ready for PrEP since more than two thirds of the women had the intention of initiating PrEP. Accordingly, program implementers should use this opportunity to expand PrEP activities nation-wide.

## Data Availability

The raw data supporting the conclusions of this article will be made available by the authors, without undue reservation.
