# Supplementary material for: Uptake of and intention to use oral pre-exposure prophylaxis for HIV among pregnant and post-natal women in Eswatini: a cross-sectional survey
Source: Front Reprod Health. 2023 Oct 27;5:1253384. doi: 10.3389/frph.2023.1253384 (PMC10641516; doi:10.3389/frph.2023.1253384)
Supplement: Supplementary file 1 [file Table1.docx]

Supplemental Table 1: Study Variables

| **Statements** | **Cronbach’s Alpha** | **Mean** | **Standard Deviation** | **Range** |
| --- | --- | --- | --- | --- |
| **PREP ATTITUDES** | | | | |
| 1. PrEP is effective at preventing HIV. 2. People who take PrEP are responsible 3. Taking PrEP is safe. 4. It would be no trouble to take PrEP every day. 5. The government makes certain that drugs like PrEP are safe.   *(Response options: 1=Strongly disagree, 2=Disagree, 3=Neutral, 4=Agree, 5=Strongly agree)* | 0.667 | 4.2 | 0.696 | 1-5 |
| **MOTIVATION TO USE PREP** | | | | |
| 1. I think I would be less worried about HIV infection if I were on PrEP 2. If I were on PrEP, I would not be concerned about the potential side-effects of PrEP 3. If I disclose that I’m on PrEP to my sex partner, he/she will be comfortable with it. 4. I have a responsibility to contribute to HIV prevention efforts by using PrEP 5. I would take PrEP if I know someone (e.g., friend, family member) who is currently taking it 6. I have family members or friends to encourage me to take PrEP properly.   *(Response options: 1=Strongly disagree, 2=Disagree, 3=Neutral, 4=Agree, 5=Strongly agree)* | 0.581 | 3.6 | 0.768 | 1-5 |
| **SELF-EFFICACY** | | | | |
| 1. How confident are you that you would stick to your PrEP medication even if you have some side-effects (e.g.? nausea) 2. How confident are you that you could make PrEP part of your daily routine? 3. How confident are you that you could get PrEP refills before you run out? 4. How confident are you that you could continue with your PrEP regimen even if getting to your clinic appointments is a major hassle? 5. How confident are you that you would use condoms while on PrEP?   *(Response options: 1=Not at all confident, 2=Slightly confident, 3=Somewhat confident, 4=Fairly confident, 5=Completely confident)* | 0.803 | 3.9 | 1.117 | 1-5 |
| **PREP STIGMA** | | | | |
| 1. If I were to use PrEP, people would think that I have HIV. 2. My friends would think less of me if they found out I was using PrEP. 3. My family would think less of me if they found out I was using PrEP. 4. People would feel uncomfortable with me if they found out that I used PrEP. 5. People would avoid me if they found out that I used PrEP. 6. If I were going to use PrEP, I would feel a need to hide that from other people. 7. If I used PrEP, I would worry that people would tell others that I am using PrEP. 8. I would worry about telling people that I take a medicine like PrEP for my health’s sake. 9. If I were to use PrEP, people would think that I have sex with a lot of different people. 10. If I were to use PrEP, people would think that I like having strange types of sex. 11. PrEP is mostly meant for people who can’t use condoms. 12. PrEP is something used mostly by people who don’t have a lot of money. 13. If I were to bring up the subject of using PrEP with my partner, he would think that I am having risky sex with other people.   *(Response options: 1= Strongly agree; 2=Agree; 3=Neutral, 4=Disagree; 5=Strongly disagree)* | 0.842 | 3.3 | 0.825 | 1-5 |
| **WILLINGNESS TO USE PREP** | | | | |
| 1. If PrEP became available, do you think you would use it? 2. Would you take PrEP as soon as it becomes available? 3. Would you take PrEP if it caused mild temporary side effects? 4. Would you take PrEP if you had to pay for it? 5. Would you take PrEP even if you have to use condoms? 6. Do you think you would use PrEP if needed to be tested regularly for HIV?   *(Response options: 1= No, definitely not; 2= No, probably not; 3= Yes, probably; 4= Yes, definitely)* | 0.836 | 3.0 | 0.744 | 1-4 |
| **INTENTION TO USE PREP** | | | | |
| 1. During the next three months, I will talk to a health care provider about PrEP. 2. During the next three months, I will seek out more information about PrEP. 3. During the next three months, I will get a prescription for PrEP.   *(Response options: 1= No, definitely not; 2= No, probably not; 3= Yes, probably; 4= Yes, definitely)* | 0.901 | 3.1 | 0.927 | 1-4 |
