# Supplementary material for: Uptake of and intention to use oral pre-exposure prophylaxis for HIV among pregnant and post-natal women in Eswatini: a cross-sectional survey
Source: Front Reprod Health. 2023 Oct 27;5:1253384. doi: 10.3389/frph.2023.1253384 (PMC10641516; doi:10.3389/frph.2023.1253384)
Supplement: Supplementary file 2 [file Table2.docx]

Supplemental Table 2: Current and preferred sources of PrEP information and pills

|  | **Where have you heard about PrEP? (N=927)** | **Where would you like to get information about PrEP? (N=1,149)** | **Where would you like to access PrEP? (N=1,149)** |
| --- | --- | --- | --- |
|  | **n (%)** | **n (%)** | **n (%)** |
| Clinic/hospital | 706 (76.2) | 1,039 (90.4) | 1,112 (96.8) |
| Community/Outreach event | 150 (16.2) | 151 (13.1) | 101 (8.8) |
| Radio | 105 (11.3) | 90 (7.8) | 5 (0.4) |
| Friend | 101 (10.9) | 21 (1.8) | 1 (.01) |
| School | 42 (4.5) | 68 (5.9) | 19 (1.7) |
| Family members | 18 (1.9) | - | - |
| None Governmental Organization | 13 (1.4) | - | - |
| Internet and Social Media; Facebook, WhatsApp | 9 (1.0) | 61 (5.3) | - |
| Workplace | 9 (1.0) | 5 (0.4) | - |
| Television | 8 (0.9) | 30 (2.6) | 2 (0.2) |
| Phone calls, email, sms | - | 13 (1.1) | - |
| Church | - | 10 (0.9) | 4 (0.3) |
| Shop/Pharmacy | - | - | 12 (1.0) |
| Automatic dispenser | - | - | 8 (0.7) |
| Other | 12 (1.3) | 22 (1.9) | 15 (1.3) |
| **Respondents allowed to mentioned more than 1 source* | | | |
