# Supplementary material for: Uptake of and intention to use oral pre-exposure prophylaxis for HIV among pregnant and post-natal women in Eswatini: a cross-sectional survey
Source: Front Reprod Health. 2023 Oct 27;5:1253384. doi: 10.3389/frph.2023.1253384 (PMC10641516; doi:10.3389/frph.2023.1253384)
Supplement: Supplementary file 3 [file Table3.docx]

Supplemental Table 3: Experiences with using PrEP among current and former PrEP users

| **Experiences with using PrEP among PrEP former users (N=102)** |
| --- |

| Questions/Statement | | Count | Percent |
| --- | --- | --- | --- |
| Last Used PrEP | | | |
|  | Less than 7 days ago | 3 | 3.3 |
|  | Less than 4 weeks ago | 5 | 5.6 |
|  | Less than 12 months ago | 70 | 77.8 |
|  | 1 and more years ago | 12 | 13.3 |
|  | Missing | 12 | - |
| Why did you discontinue taking PrEP pills? | | | |
|  | Side Effects | 32 | 35.2 |
|  | Other reasons | 59 | 64.8 |
|  | Missing | 11 | - |
| Specify other reason for stopping PrEP | | | |
|  | PrEP not available at health facility/ stock outs/ ran out of PrEP pills | 15 | 27.8 |
|  | Stopped being at risk/Felt did not need PrEP anymore | 7 | 13.0 |
|  | Stopped by partner/husband | 6 | 11.1 |
|  | Struggled to take PrEP pills on time | 6 | 11.1 |
|  | Got pregnant was afraid of side effects/got sick during pregnancy | 5 | 9.3 |
|  | Not knowledgeable about PrEP/Didn’t know had to refill | 4 | 7.4 |
|  | Transferred to different health facility too far | 3 | 5.6 |
|  | Opted to use condoms instead | 2 | 3.7 |
|  | Discouraged by a nurse | 1 | 1.9 |
|  | Lengthy process within health facility to get PrEP pills | 1 | 1.9 |
|  | Prefer an injection instead of pills | 1 | 1.9 |
|  | Tablet too big to swallow | 1 | 1.9 |
|  | Had people talk negatively about PrEP and stopped | 1 | 1.9 |
|  | Just got demotivated to take PrEP | 1 | 1.9 |
|  | Too much medication/pill burden | 0 | 0.0 |
|  | Missing | 5 | - |
| Side effects experienced when taking PrEP? | | | |
| *Nausea* | | | |
|  | Resolved | 8 | 26.7 |
|  | Ongoing | 5 | 16.7 |
|  | Not experienced | 17 | 56.7 |
|  | Missing | 2 |  |
| *Vomiting* | | | |
|  | Resolved | 12 | 40.0 |
|  | Ongoing | 2 | 6.7 |
|  | Not experienced | 16 | 53.3 |
|  | Missing | 2 |  |
| *Fatigue* | | | |
|  | Resolved | 4 | 13.3 |
|  | Ongoing | 3 | 10.0 |
|  | Not experienced | 23 | 76.7 |
|  | Missing | 2 |  |
| *Dizziness* | | | |
|  | Resolved | 11 | 36.7 |
|  | Ongoing | 5 | 16.7 |
|  | Not experienced | 14 | 46.7 |
|  | Missing | 2 |  |
| *Headache* | | | |
|  | Resolved | 7 | 23.3 |
|  | Ongoing | 5 | 16.7 |
|  | Not experienced | 18 | 60.0 |
|  | Missing | 2 |  |
| *Rash* | | | |
|  | Resolved | 1 | 3.3 |
|  | Ongoing | 2 | 6.7 |
|  | Not experienced | 27 | 90.0 |
|  | Missing | 2 |  |
| *Abdominal pain* | | | |
|  | Resolved | 4 | 13.3 |
|  | Ongoing | 2 | 6.7 |
|  | Not experienced | 24 | 80.0 |
|  | Missing | 2 |  |
| *Weight loss* | | | |
|  | Resolved | 2 | 6.7 |
|  | Ongoing | 0 | 0.0 |
|  | Not experienced | 28 | 93.3 |
|  | Missing | 2 |  |
| Would you like to start taking PrEP again? | | | |
|  | No | 40 | 39.2 |
|  | Yes | 62 | 60.8 |
| Reasons for resuming to take PrEP pills? | | | |
|  | To protect myself from getting infected with HIV | 23 | 42.6 |
|  | Do not trust partner | 8 | 14.8 |
|  | Can take PrEP again if needed/feeling at risk | 5 | 9.3 |
|  | PrEP is important/helpful/It is a responsible thing to do | 4 | 7.4 |
|  | Will stop breastfeeding soon | 3 | 5.6 |
|  | Now knowledgeable about/understands PrEP | 3 | 5.6 |
|  | PrEP is now available in the health facility | 2 | 3.7 |
|  | Partner is HIV positive/On ART | 2 | 3.7 |
|  | I am sexually active again | 2 | 3.7 |
|  | Have delivered baby, perceive less threat/side effects to baby | 1 | 1.9 |
|  | To protect baby form acquiring HIV | 1 | 1.9 |
|  | Does not know partner’s HIV Status | 0 | 0.0 |
|  | Missing | 8 | - |
|  |  |  |  |
| Experiences with taking PrEP among current PrEP users (N=183) | | | |

| Questions/Statement | | Count | | Percent |
| --- | --- | --- | --- | --- |
| In the last 3 months, have you experienced any side effects as a result of taking PrEP? | | | | |
|  | No | | 120 | 65.6 |
|  | Yes | | 63 | 34.4 |
| Side effects experienced when taking PrEP? | | | | |
| *Nausea* | | | | |
|  | Resolved | | 12 | 19.4 |
|  | Ongoing | | 13 | 21.0 |
|  | Not experienced | | 37 | 59.7 |
|  | Missing | | 1 | - |
| *Vomiting* | | | | |
|  | Resolved | | 15 | 24.2 |
|  | Ongoing | | 4 | 6.5 |
|  | Not experienced | | 43 | 69.4 |
|  | Missing | | 1 | - |
| *Fatigue* | | | | |
|  | Resolved | | 12 | 19.4 |
|  | Ongoing | | 6 | 9.7 |
|  | Not experienced | | 44 | 71.0 |
|  | Missing | | 1 | - |
| *Dizziness* | | | | |
|  | Resolved | | 22 | 35.5 |
|  | Ongoing | | 12 | 19.4 |
|  | Not experienced | | 28 | 45.2 |
|  | Missing | | 1 | - |
| *Headache* | | | | |
|  | Resolved | | 18 | 29.0 |
|  | Ongoing | | 8 | 12.9 |
|  | Not experienced | | 36 | 58.1 |
|  | Missing | | 1 | - |
| *Rash* | | | | |
|  | Resolved | | 2 | 3.2 |
|  | Ongoing | | 3 | 4.8 |
|  | Not experienced | | 57 | 91.9 |
|  | Missing | | 1 | - |
| *Abdominal pain* | | | | |
|  | Resolved | | 4 | 6.5 |
|  | Ongoing | | 7 | 11.3 |
|  | Not experienced | | 51 | 82.3 |
|  | Missing | | 1 | - |
| *Weight loss* | | | | |
|  | Resolved | | 1 | 1.6 |
|  | Ongoing | | 7 | 11.3 |
|  | Not experienced | | 54 | 87.1 |
|  | Missing | | 1 | - |
| Please rate your ability, over the past month, to take pills exactly as you were instructed | | | | |
|  | Very poor | | 1 | .5 |
|  | Poor | | 3 | 1.6 |
|  | Fair | | 28 | 15.3 |
|  | Good | | 41 | 22.4 |
|  | Very good | | 52 | 28.4 |
|  | Excellent | | 58 | 31.7 |
| Does taking pills interfere with any of your regular daily activities? | | | | |
|  | No | | 162 | 89.0 |
|  | Yes | | 20 | 11.0 |
|  | Missing | | 1 | - |
| In the past month, what time of day did you typically take your pill? | | | | |
|  | Morning | | 82 | 45.1 |
|  | Afternoon | | 12 | 6.6 |
|  | Evening | | 87 | 47.8 |
|  | Other | | 1 | .5 |
|  | Missing | | 1 | - |
| In the past month, how often did you take your pill at about the same time each day? | | | | |
|  | Never | | 7 | 3.8 |
|  | Sometimes | | 56 | 30.8 |
|  | Always | | 119 | 65.4 |
|  | Missing | | 1 | - |
| In the past month, what has helped you remember to take your pill? | | | | |
|  | Alarm/cell phone | | 88 | 48.1 |
|  | Nothing | | 46 | 25.1 |
|  | Association with a daily activity | | 21 | 11.5 |
|  | Husband/boyfriend/ primary partner | | 19 | 10.4 |
|  | Family member or friend | | 13 | 7.1 |
|  | Association with taking other pills or medication | | 12 | 6.6 |
|  | Pill box | | 7 | 3.8 |
|  | Calendar | | 1 | .5 |
|  | Association with having sex | | 0 | 0.0 |
|  | Other | | 16 | 8.7 |
| Who have you disclosed that "you are taking PrEP medication?" | | | | |
|  | Husband/Boy friend | | 142 | 77.6 |
|  | Mother or father | | 91 | 49.7 |
|  | Other family member | | 54 | 29.5 |
|  | Friends | | 18 | 9.8 |
|  | Children | | 13 | 7.1 |
|  | No one | | 10 | 5.5 |
|  | Neighbors | | 4 | 2.2 |
|  | Other | | 3 | 1.6 |
| Please tell me all of the reasons that kept you from taking your pill. | | | | |
|  | I forgot or was too busy | | 24 | 13.2 |
|  | I did not have pill with me | | 20 | 11.0 |
|  | I ran out of or lost pills | | 7 | 3.8 |
|  | I got tired of taking a pill everyday | | 4 | 2.2 |
|  | I had a change in daily routine | | 3 | 1.6 |
|  | I had side effects from pills | | 2 | 1.1 |
|  | I felt sick/was concerned about getting sick | | 1 | .5 |
|  | My husband/ boyfriend/primary partner did not approve of me taking the pill | | 1 | .5 |
|  | Other | | 10 | 5.5 |
| In the past month, how often did you take more than one pill in a day? | | | | |
|  | Never | | 167 | 92.8 |
|  | Sometimes | | 8 | 4.4 |
|  | Always | | 5 | 2.8 |
|  | Missing | | 3 | - |
| In the past month, how many of your pills did you give away, exchange, trade or sell? | | | | |
|  | None of them | | 180 | 99.4 |
|  | Some of them | | 1 | .6 |
|  | Most of them | | 0 | 0.0 |
|  | All of them | | 0 | 0.0 |
|  | Missing | | 2 | - |
| Who did you give away, exchange, trade, or sell your pills to? | | | | |
|  | Husband/ boyfriend/ primary partner | | 0 | 0.0 |
|  | Another sex worker | | 0 | 0.0 |
|  | Family member/ relative | | 0 | 0.0 |
|  | Friend | | 1 | .5 |
|  | Other | | 0 | 0.0 |
| Do you want to continue taking PrEP for the next month? | | | | |
|  | No | | 8 | 4.4 |
|  | Yes | | 174 | 95.6 |
| Reasons for not wanting to continue taking PrEP | | | | |
|  | I will not be having sex/ no longer at risk | | 4 | 50.0 |
|  | I do not like taking PrEP | | 0 | 0.0 |
|  | I get side effects from taking PrEP | | 0 | 0.0 |
|  | I find it too difficult to remember to take PrEP | | 0 | 0.0 |
|  | Will stop breast feeding | | 1 | 12.5 |
|  | Tired of taking pills everyday | | 1 | 12.5 |
|  | Need a break | | 1 | 12.5 |
|  | No response | | 1 | 12.5 |
